# Direct visualization of both DNA and RNA quadruplexes in human cells *via* an uncommon spectroscopic method

**DOI:** 10.1038/srep32141

**Published:** 2016-08-18

**Authors:** Aurélien Laguerre, Judy M. Y. Wong, David Monchaud

**Affiliations:** 1 Institut de Chimie Moléculaire, ICMUB CNRS UMR6302, UBFC, 21078 Dijon, France; 2 Faculty of Pharmaceutical Sciences, The University of British Columbia, Vancouver, Canada

## Abstract

Guanine-rich DNA or RNA sequences can fold into higher-order, four-stranded structures termed quadruplexes that are suspected to play pivotal roles in cellular mechanisms including the control of the genome integrity and gene expression. However, the biological relevance of quadruplexes is still a matter of debate owing to the paucity of unbiased evidences of their existence in cells. Recent reports on quadruplex-specific antibodies and small-molecule fluorescent probes help dispel reservations and accumulating evidences now pointing towards the cellular relevance of quadruplexes. To better assess and comprehend their biology, developing new versatile tools to detect both DNA and RNA quadruplexes in cells is essential. We report here a smart fluorescent probe that allows for the simple detection of quadruplexes thanks to an uncommon spectroscopic mechanism known as the red-edge effect (REE). We demonstrate that this effect could open avenues to greatly enhance the ability to visualize both DNA and RNA quadruplexes in human cells, using simple protocols and fluorescence detection facilities.

Quadruplexes are four-stranded DNA or RNA structures that fold from guanine-rich sequences and are stabilized with the formation of interstrand guanine quartets (Fig. 1)^1^,^2^. Quadruplexes are implicated in pivotal metabolic processes involving nucleic acids, including the regulation of transcription, translation and replication^3^. Consequently, these alternative nucleic acid structures are intensively studied for their roles in genetic dysfunctions^4^, as therapeutic targets in cancers^5^ and neurodegenerative^6^, viral or other infectious diseases^7^. Owing to their transient formation and resolution in genome and transcriptome, the design of tools to assess whether, where and when quadruplexes form in eukaryotic cells is challenging^8^,^9^. First evidences of the existence of quadruplexes in cells were provided by immunodetection studies^10^,^11^,^12^,^13^,^14^, and by *in situ* fluorescence labelling of quadruplex ligands^15^. The recent development of quadruplex-selective fluorophores^16^ was critical since only fluorescent small molecules allow for the visualization of quadruplexes in live cells^17^,^18^,^19^,^20^, contrary to the antibody-based approaches that are limited to fixed and permeabilized cells.

NaphthoTASQ (or N-TASQ, TASQ standing for template-assembled synthetic G-quartet, Fig. 1) is one of these probes^18^. It belongs to the family of twice-as-smart ligands, being both a smart quadruplex ligand and a smart fluorescent probe^21^. Upon interaction with quadruplexes, it undergoes a structural switch that triggers both its affinity (smart ligand) and its fluorescence (smart probe). N-TASQ interacts with quadruplexes *via* a biomimetic approach based on the self-assembly of quartets, one from the quadruplex and one from the ligand^22^,^23^. Theoretically, a spectroscopic limitation of the use of N-TASQ lies in its inability to absorb light at wavelengths above 320 nm, therefore precluding the use of standard epifluorescence and confocal microscopes (most commonly equipped with lasers adjusted at 408, 488 and 555 nm) for cell imaging. Consequently, N-TASQ was used in the tracking quadruplexes in cells with two-photon microscopy^18^.

Herein, we discovered that N-TASQ was also compatible with confocal imaging, and determined the ligand’s unique spectroscopic property known as the red-edge effect (REE). We demonstrated the versatility of N-TASQ as a REE probe with confocal microscopy and reported on the convenient visualization of both DNA and RNA quadruplexes in human cells.

## Results and Discussion

### The N-TASQ structure and the red-edge effect

**Figure 1:**
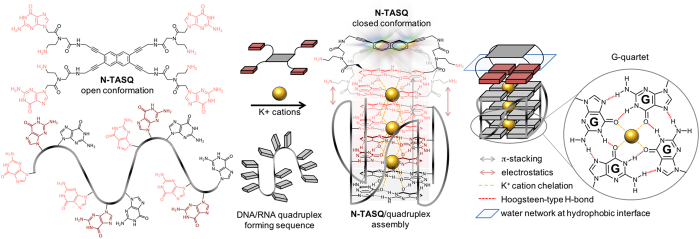
Bioinspired recognition of quadruplexes by synthetic G-quartets. Structure of a template-assembled synthetic G-quartet (or TASQ, here N-TASQ, *left*) and of a G-quartet (*right*); schematic representation of a quadruplex-forming sequence in its unfolded and folded states. The bioinspired interaction between N-TASQ and quadruplexes takes place through quartet-quartet recognition, stabilized by π-stacking interactions (grey arrows) and cation chelation (yellow dashed lines), along with electrostatic interactions (pink arrows). The ordered hydration shell of the TASQ/quadruplex assembly is schematically represented as a pale blue rectangle.

The N-TASQ structure comprises a fluorogenic naphthalene template surrounded by four synthetic guanine arms, built from ^PNA^G (peptidic nucleic acid guanine) monomers. When free in solution, N-TASQ adopts an ‘open’ conformation (Fig. 1) in which the four electron-rich guanines restrain the fluorescence of the naphthalene template, making N-TASQ non-fluorescent. In contrast, upon interaction with its quadruplex targets, N-TASQ folds into a ‘closed’ conformation (Fig. 1) in which its four guanines form an intramolecular G-quartet, promoted by interaction (stacking) with the accessible G-quartets of the quadruplexes in a bioinspired manner. The electronic density of guanines embedded in a G-quartet decreases as compared to free guanines due to electron redistribution triggered by quartet formation^24^, thereby relieving the template from its electronic constraint. Thus, N-TASQ becomes fluorescent upon interaction with quadruplexes, making it a quadruplex-selective smart fluorescent probe.

By chemical design, N-TASQ absorbs light below 320 nm only. This restriction precludes the use of confocal microscopes as they are commonly equipped with lasers adjusted at longer wavelengths (usually at 408, 488 and 555 nm). However, a recent report on the peculiar spectroscopic properties displayed by polymeric ^PNA^G assemblies lead us to reconsider the optical features of N-TASQ. Gazit *et al.* reported that ^PNA^G assemblies can emit fluorescence without added dye, in a excitation-dependent manner under virtually any excitation wavelength^25^. This peculiar spectroscopic phenomenon is known as the red-edge effect (REE)^26^. In essence (the full explanation of this effect can be found in the Supplementary Material), upon strong interaction between two aromatic partners (in this case, two ^PNA^G), the water molecule motions in the hydration shell of the resulting assemblies are slowed down considerably, giving rise to various intermediate fluorogenic systems that could be excited by photons of lower energy, that is, shifted toward the long wavelength edge (the so-called red-edge) of their absorption bands. REE offers the possibility of exciting the assembled partners far beyond the limits defined by each member’s respective UV-Vis spectra. We hypothesize that N-TASQ/quadruplex complex (naphthalene/^PNA^G/G assembly) could be considered as another ^PNA^G assembly with corresponding REE. If our assertion is proven correct, it would offer a unique opportunity to use N-TASQ for quadruplex cellular imaging with confocal microscopy.

### The demonstration of the red-edge effect

**Figure 2:**
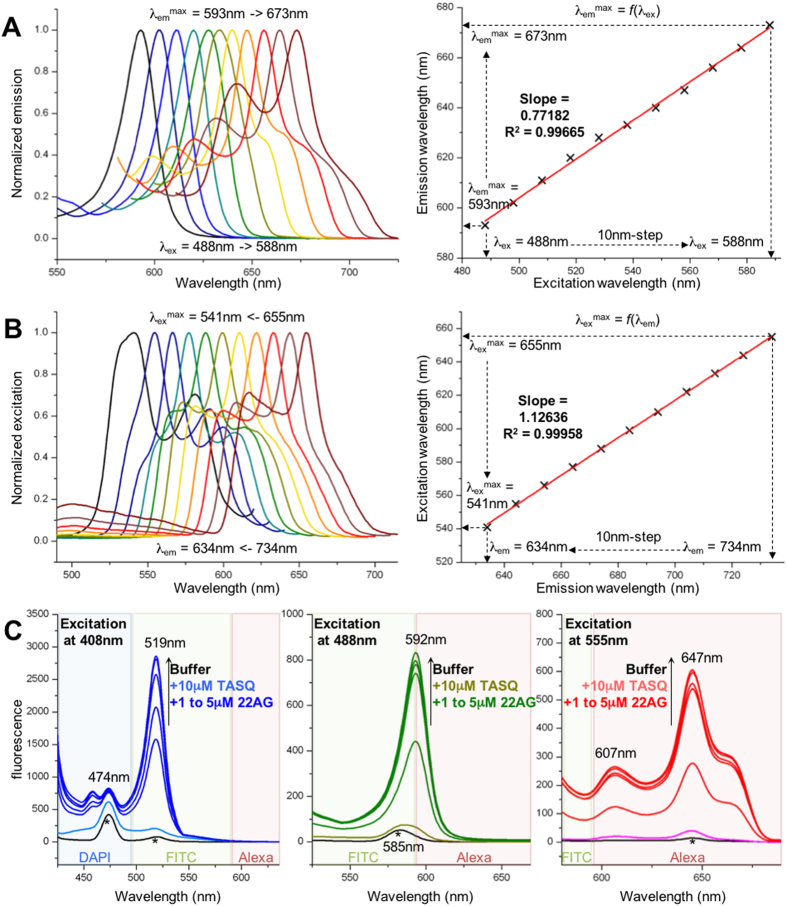
Demonstration and exploitation of the red-edge effect. (**A**) Dependence of the emission maximum (λ_em_^max^) on the excitation wavelength (λ_ex_): fluorescence emission spectra recorded for an experiment carried out with N-TASQ (10 μM) and a quadruplex (22AG, 5 μM) with λ_ex_ between 488 and 588 nm (every 10 nm). (**B**) Dependence of the excitation maximum (λ_ex_^max^) on the emission wavelength (λ_em_): fluorescence excitation spectra recorded for an experiment carried out with N-TASQ (10 μM) and a quadruplex (22AG, 5 μM) with λ_em_ between 734 and 634 nm (every 10 nm). (**C**) Fluorescence titrations performed without (black lines) or with N-TASQ (10 μM) and increasing amounts of 22AG (from 0 to 5 μM) with λ_ex_ at 408 (left), 488 (center) and 555 nm (right). Black stars indicate the Raman signals of the buffer. All experiments are performed in 10 mM lithium cacodylate buffer (pH 7.2) + 10 mM KCl/90 mM LiCl.

The REE, known since 1970^27^,^28^, primarily concerns material sciences since highly organized media (vitrified glasses, dense polymeric matrices, etc.) are particularly prone to this optical feature. Life science is also a venue for REE, notably in proteins science in which tryptophan residues deeply buried in the hydrophobic binding pocket of proteins serve as REE probes for monitoring protein structural changes and ligand binding events^29^. As a distinctive signature, REE is characterized by a dependence of the wavelength of the emission maximum on the excitation wavelength, λ_em_^max^ = *f*(λ_ex_), and by the reciprocal dependence of the wavelength of the excitation maximum on the emission wavelength, λ_ex_^max^ = *f*(λ_em_). We thus investigated whether N-TASQ in interaction with a quadruplex-DNA (here 22AG, d[^5′^AG_3_(T_2_AG_3_)_3_^3′^], which mimics the human telomeric sequence)^30^ could *i*- fluoresce when excited at wavelengths beyond 320 nm (for instance 488 nm and above) and *ii*- display emission wavelengths (λ_em_^max^) dependent on the excitation wavelengths (λ_ex_). *In vitro* experiments were carried out with N-TASQ (10 μM) and 22AG (5 μM), and our data showed that not only the corresponding assembly fluoresces when irradiated at 488 nm and above, but also that the correlation between λ_ex_ (we performed serial excitations with a range of wavelengths every 10 nm between 488 and 588 nm) and λ_em_^max^ (from 593 to 673 nm) is linear, as demonstrated by the strong regression R^2^ = 0.99665 (Fig. 2A). Of note, the slope of the fitted data is 0.77182, which highlights that Stokes shift gradually decreases (from 105 to 85 nm) when the λ_ex_ increases towards the red region. The Stokes shift, which is the difference between the excitation and emission bands, is mostly due to solvent relaxation: conceivably, longer wavelengths (*i.e.*, lower energy) imply lower energy difference due to solvent relaxation, thus contributing to lower Stokes shifts. We also demonstrated that the reciprocal relationship between wavelengths of emission (λ_em_, every 10 nm between 734 and 634 nm) and excitation (λ_ex_^max^, from 655 to 541 nm) is also linear, with a very strong regression (R^2^ = 0.99958) and a slope of 1.12636 that again illustrated the dynamic Stokes shifts (from 79 to 93 nm when the λ_em_ decreases towards the blue region, Fig. 2B). Together, this series of data demonstrated that N-TASQ/quadruplex assemblies exhibited REE and could be visualized by various fluorescence detection facilities including routine confocal laser microscopy.

To further support this notion, the fluorescence emission of N-TASQ/quadruplex assemblies following irradiation at wavelengths corresponding to common confocal laser excitation (*i.e.*, 408, 488 and 555 nm) was quantified. Serial titrations were carried out for each excitation wavelength with 10 μM N-TASQ and increasing amounts of 22AG (from 0 to 5 μM): the emission patterns observed (Fig. 2C, with maxima at 474, 519, 592, 607 and 647 nm) are completely compatible with standard emission filters routinely used in confocal microscopes (DAPI: 495 nm and below; AF488, or FITC: 495–590 nm; and AF594, or Alexa: 585 nm and above, schematically represented as colored areas in Fig. 2C). These results again demonstrate that N-TASQ is suited to confocal imaging analyses. To assess whether REE originates from the restriction of the rotational mobility of sequestered water molecules by aromatic interfaces^31^,^32^, the modification of the water’s Raman signals^33^,^34^ upon addition of TASQ and then DNA was monitored. The fluorescence spectra of buffer alone (10 mM lithium cacodylate, pH 7.2, plus 90 mM LiCl/10 mM KCl) was used to determine the Raman signals of water (474/519, 585 and 645 nm for an excitation at 408, 488 and 555 nm, respectively, black lines and stars, Fig. 2C). Upon addition of N-TASQ (10 μM) followed by increasing amounts of quadruplex (from 1 to 5 μM), the Raman signals of water increase, thus reflecting the modification of ordered water molecules in the hydration shell of the TASQ/quadruplex assembly.

**Figure 3:**
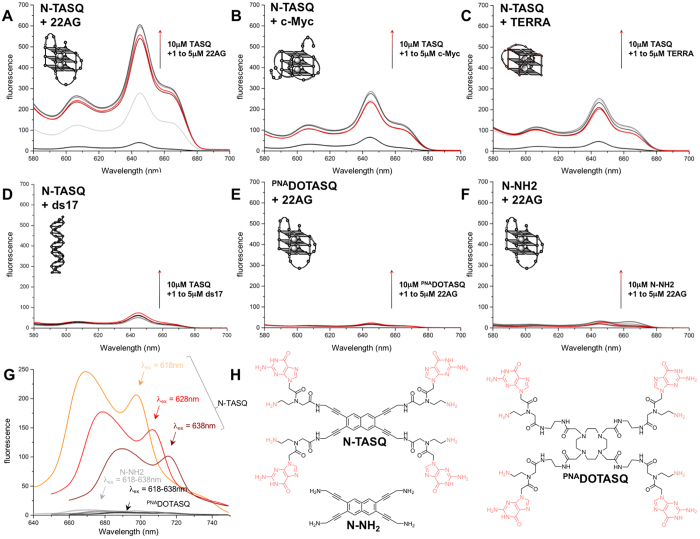
Assessing the properties of N-TASQ as quadruplex-specific REE probe. (**A–D**) Fluorescence titrations performed without (black lines) or with N-TASQ (10 μM) and increasing amounts of oligonucleotides (from 0 to 5 μM, from grey to red lines), either quadruplex-DNA (22AG, (**A)** or c-Myc, (**B**)), quadruplex-RNA (TERRA, (**C**)) or duplex-DNA (ds17, (**D**)). (**E,F)** control experiments carried out without (black lines) or with 10 μM of ^PNA^DOTASQ (**E**) or N-NH_2_ (**F**) and increasing amounts of 22AG (from 0 to 5 μM, from grey to red lines). All experiments are performed under excitation (λ_ex_) at 555 nm. (**G)** REE experiments performed with N-TASQ (colored lines), ^PNA^DOTASQ (black lines) or N-NH_2_ (grey lines) and 22AG (5 μM) with λ_ex_ at 618, 628 and 638 nm. All experiments are performed in 10 mM lithium cacodylate buffer (pH 7.2) + 10 mM KCl/90 mM LiCl. (**H**) structures of N-TASQ, ^PNA^DOTASQ and N-NH_2_.

To confirm that N-TASQ is a unique quadruplex-specific REE probe, a series of control experiments was performed (Fig. 3 and the Supplementary Material, Figures S3 and S4): first, we demonstrated that REE could be observed with N-TASQ binding to a variety of quadruplex-forming sequences, including the quadruplex-DNA 22AG (Fig. 3A, see above) and c-Myc (d[^5′^GAG_3_TG_4_AG_3_TG_4_A_2_G^3′^], which mimics the sequence found in the promoter region of the *myc* gene, Fig. 3B)^5^, as well as the quadruplex-RNA TERRA (r[^5′^G_3_(U_2_AG_3_)_3_^3′^], which corresponds to telomeric RNA transcripts, Fig. 3C)^35^,^36^; second, we showed that N-TASQ did not fluoresce when duplex-DNA was added under these conditions (ds17, d[^5′^C_2_AGT_2_C(GTA)_2_AC_3_^3′^]/d[^5′^G_3_T(TAC)_2_GA_2_CTG_2_^3′^], Fig. 3D); and third, we comparatively studied the putative REE properties of two compounds closely related to N-TASQ, namely ^PNA^DOTASQ (a TASQ devoid of fluorogenic template, Fig. 3E,H)^23^ and N-NH2 (a N-TASQ derivative devoid of guanine arms, Fig. 3F,H)^18^: both compounds could not trigger REE responses (Fig. 3E–G), thus confirming that both guanine (for biomimetic interactions with quadruplexes) and naphthalene (as polarizable aromatic dye) moieties are mandatory to induce REE. Collectively, our results show that N-TASQ is a unique quadruplex-specific REE probe for confocal analyses.

### DNA, RNA quadruplexes and cellular imaging

**Figure 4:**
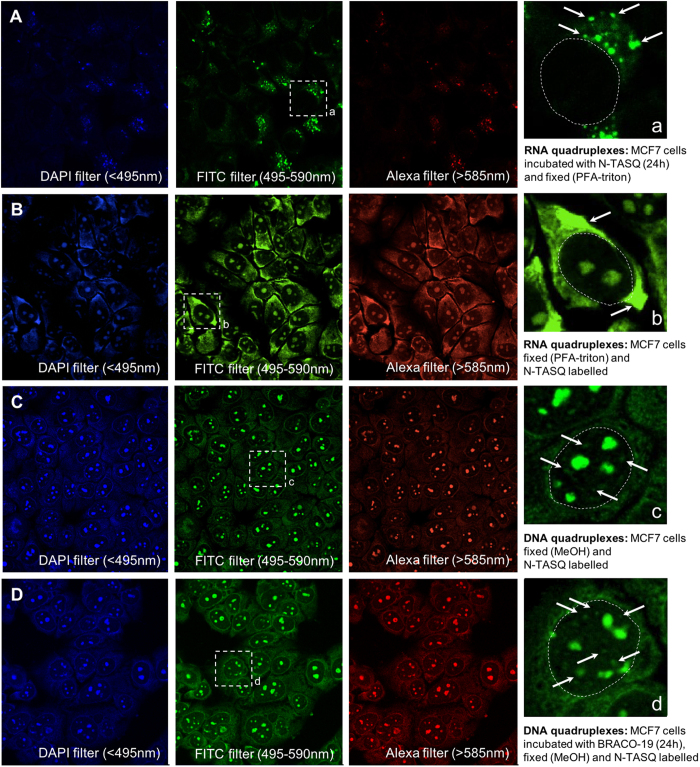
Implementation of the red-edge effect for fluorescence cell imaging. (**A**) Images of MCF7 cells live-treated with N-TASQ (2.5 μM) for 24 h before fixation (PFA-triton). (**B**) Images of fixed MCF7 cells (PFA-triton) post-labelled with N-TASQ (100 μM). (**C**) Images of fixed MCF7 cells (MeOH) post-labelled with N-TASQ (100 μM). (**D**) Images of MCF7 cells live-treated with BRACO-19 (2.5 μM), followed by incubation with N-TASQ (100 μM) for 1 h after cell fixation (MeOH). Fixed cells are mounted (Fluoromount-G^TM^) for confocal analyses carried out with lasers at 408, 488 and 555 nm and visualized through DAPI (blue), FITC (green) and Alexa channels (red). White arrows in insets indicate representative clusters of RNA (**A,B**) and DNA (**C,D**) quadruplexes.

N-TASQ was previously used for tracking RNA quadruplexes in live cells *via* multiphoton microscopy^18^. To further demonstrate its versatility, we assess here whether N-TASQ could report both RNA and DNA quadruplexes *via* confocal microscopy. Live incubation of human cells with N-TASQ (2.5 μM) diverts RNA from normal cellular processes and triggers the accumulation of RNA quadruplexes in cytoplasmic loci, enabling the detection of higher-order TASQ/quadruplex-RNA assemblies *via* two-photon microscopy. To investigate whether similar images could be collected with a confocal microscope, MCF7 cells were first incubated for 24 h with a sublethal N-TASQ concentration (2.5 μM, the cytotoxicity data can be found as Supplementary Material, Figure S5) before fixation (PFA-triton) and mounting steps required for collecting images via confocal analyses: as seen in Fig. 4A, high-resolution images were obtained using three sets of filters, with better quantum gain through the set of FITC filter, in line with the schematic presentation used for the *in vitro* binding experiments shown in Fig. 2C. Collected images highlighted the cytoplasmic distribution of intense fluorescent foci, in agreement with our previous observations; better visualization of the cytoplasmic naphthalene/^PNA^G/G assemblies was included in inset a, with a staining pattern that presumably corresponds to clusters of RNA quadruplexes (as previously demonstrated by RNase digestion studies)^18^. We also attempted to visualize RNA quadruplexes *via* a simpler protocol, labelling cells with N-TASQ (100 μM) after cell fixation (PFA-triton). Higher concentrations of N-TASQ (100 *versus* 2.5 μM) were used to optimize quadruplex detection, since post-fixation labelling relieves cytotoxicity constraints encountered during live cell incubation. As seen in Fig. 4B, this treatment provides lower contrast images, with a strong nucleoli labelling (known to be rich in quadruplex-forming sequences)^37^, along with a diffuse cytoplasmic labelling. Collectively, this series of images confirms the suitability of N-TASQ for confocal analyses, and enlighten also that the protocol based on N-TASQ live incubation followed by cell fixation, even though less straightforward, provides a better way to visualize RNA quadruplexes in fixed cells.

We next decided to investigate the influence of the fixing agent onto the labelling pattern of N-TASQ. To this end, cells were labelled with N-TASQ (100 μM) after cell fixation as above, using MeOH as fixative. As seen in Fig. 4C, collected images were not only of higher resolution but they also display distinctive features notably a very low cytoplasmic labelling along with discrete nuclear fluorescent foci around strongly stained nucleoli. For the sake of comparison with previous live-incubation experiments, images were also collected with cells post-labelled with 2.5 μM N-TASQ only: as seen in the Supplementary Material (Figure S6), a similar labelling pattern is obtained, albeit with lower overall fluorescence intensity and cytoplasm/nucleus contrast, thus bolstering the efficiency of N-TASQ as REE probe for cellular imaging but also indicating that high concentrations of N-TASQ should be preferred for obtaining high-quality images under these conditions. We then decided to assess whether the discrete nuclear foci seen in Fig. 4C (highlighted in the inset c) might correspond to DNA quadruplexes. To this end, we referred to one of the most compelling piece of evidence for the formation of DNA quadruplexes in human cells reported by Rodriguez *et al.*^15^: in this approach, live cell incubation of a quadruplex-promoting ligand (PDS-α) was followed by the visualization of the ligand/quadruplex assemblies by a chemical labelling step (*in situ* click chemistry), resulting in the covalent binding of an Alexa fluorophore to the DNA-bound ligand. This two-step approach leads to a strong labelling of nucleoli (as above) along with discrete nuclear foci, attributed to clusters of DNA quadruplexes through co-localization experiments performed with a fluorescent quadruplex-selective helicase (hPif1). We thus followed a similar approach, live-incubating MCF7 cells with a firmly established (and commercially available) quadruplex-DNA ligand BRACO-19 (2.5 μM)^38^, using N-TASQ as a post-staining agent (100 μM) after cell fixation (MeOH). As seen in Fig. 4D, we found that a high N-TASQ concentration (100 μM) provides high resolution images again but does not allow for seeing significant differences between untreated and BRACO-19-treated cells (insets c and d, Fig. 4C,D for instance). This observation might originate in a lower BRACO-19 efficiency (as compared to PDS-α) to promote the formation of genomic quadruplexes or because the two ligands (BRACO-19 and N-TASQ) target the same binding sites and are mutually exclusive. To further investigate this, we performed a series of experiments with lower concentrations of N-TASQ (2.5 μM, as post-fixation agent, see above) and increasing amounts of BRACO-19 (0–5 μg/mL in live-cell incubation experiments for 48 h, *i.e.*, 0–8.4 μM, see the Supplementary Material). Quite satisfyingly, an increase of nuclear foci was clearly observed in cells treated with low BRACO-19 concentration (0.625 μg/mL, Figure S7) over untreated cells, thus strongly supporting that N-TASQ targets quadruplex-DNA. Higher BRACO-19 concentrations (1.25–5 μg/mL) lead to a BRACO19-dose-dependent reduction of N-TASQ staining, presumably because of the competition that might take place between the two quadruplex ligands. Collectively, these series of images confirm the suitability of N-TASQ for confocal analyses and highlight the versatility of N-TASQ since it might allow for the direct visualization of DNA quadruplexes in fixed cells.

**Figure 5:**
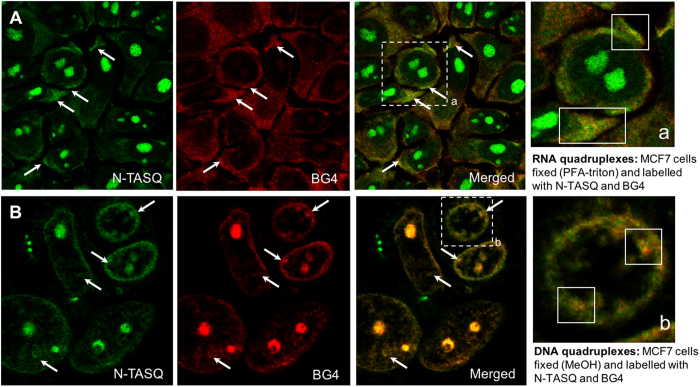
Co-incubation of BG4 and N-TASQ. (**A**,**B**) MCF7 cells are fixed either with PFA-triton (**A**) or MeOH (**B**) and labelled with BG4 (10 μg/mL) for 3 h (followed by secondary and tertiary antibodies, see the Experimental Section) and then N-TASQ (100 μM) for 3 h before mounting steps (Fluoromount-G^TM^) and confocal analyses carried out with lasers at 408, 488 and 555 nm and visualized through DAPI (blue), FITC (green) and Alexa channels (red). White arrows indicate some of the most representative N-TASQ/BG4 co-staining sites, *i.e*., clusters of RNA (**A**) or DNA (**B**) quadruplexes.

To further demonstrate the quadruplex identity of labeled N-TASQ cellular targets, a series of co-labelling experiments was performed with the quadruplex-specific antibody BG4 (preparation procedure can be found as Supplementary Material, Figure S8): this antibody can bind to both DNA and RNA quadruplexes, and the binding efficiency is dependent on the accessibility of the cellular targets and determined by the conditions of the immunodetection procedures. We found^18^ that BG4 labels quadruplexes mostly in cytoplasmic sites (RNA quadruplexes)^12^ after PFA-triton fixation and in nuclear sites (DNA quadruplexes)^11^ after MeOH fixation. We used these two conditions with MCF7 cells and post-labeled the fixed cells with both BG4 and N-TASQ. The comparison of the two corresponding labelling patterns might be complicated by the fact that N-TASQ provides fluorescence outputs in every emission filters (DAPI, FITC and Alexa), as seen above. The secondary antibody (2Ab) used to label BG4 is itself recognized by a tertiary antibody conjugated to an Alexa594 dye (AF594-3Ab), which is consequently detected through the Alexa filter, thus colliding with N-TASQ emission. However, the red-edge nature of the N-TASQ fluorescence signals make them weak at this wavelength (Fig. 2C), *a fortiori* far weaker than Alexa594 irradiated at *ad hoc* wavelength. This was demonstrated by a series of fixed cells images (with both PFA-titron and MeOH), post-labeled by either N-TASQ, BG4 or both (see the Supplementary Material, Figure S9). These images show that while both N-TASQ and BG4 provide signals in the Alexa594 channel, the fluorescence of BG4 clearly outweighs that of N-TASQ when both markers are co-incubated. This was further substantiated by a series of control experiments (see the Supplementary Material, Figure S10) performed with fixed cells labeled with a/N-TASQ, b/N-TASQ and BG4/2Ab/AF594-3Ab, and c/N-TASQ and 2Ab/AF594-3Ab. Therefore, the comparison between N-TASQ and BG4 patterns can be reliably made through the blue and green channels (where only N-TASQ is fluorescent) and red channels (where BG4 fluorescence prevails). As seen in Fig. 5A, PFA-triton fixation followed by co-incubation with BG4 and N-TASQ results in the labelling of both cytoplasmic sites and nucleoli by N-TASQ (green channel) and of cytoplasmic sites only by BG4 (red channel); merging the two images highlights co-localization foci (white arrows and inset a), thus indicating that both tools target the same in cytoplasmic sites. Conversely, as seen in Fig. 5B, MeOH fixation followed by co-incubation with BG4 and N-TASQ results in the labelling of nucleoli and sub-nuclear sites by both N-TASQ (green channel) and BG4 (red channel); merging the two images highlights co-localization foci (white arrows and inset b), which again indicates that both tools target the same nuclear sites. We also collected images upon the aforementioned experimental setup, live-incubating cells with BRACO-19 (0.625 μg/mL) and labelling them with N-TASQ (2.5 μM) after MeOH fixation –a protocol previously demonstrated to allow for convenient quadruplex visualization–, adding BG4 incubation as an extra step. Results seen in Figure S10 first confirm that the images collected through the red channel is reflective of BG4 labelling only (and consequently that blue and green channels are reflective of N-TASQ labelling) but also indicate that BG4 incubation precludes efficient N-TASQ staining when it is used at low concentrations (2.5 μM) due to a competition between the antibody and the ligand. Collectively, these experiments demonstrate that N-TASQ and BG4 can be used concomitantly and that they have similar cellular targets, therefore lending credence to the capability of N-TASQ to detect both DNA and RNA quadruplexes in fixed cells.

## Discussion

We have demonstrated here that N-TASQ could represent a new generation of quadruplex markers, the quadruplex-specific red-edge effect (REE) probes, which is remarkable due to its versatility for cellular imaging as it uniquely allows for *i*- tracking both DNA and RNA quadruplexes, *ii*- in both live and fixed human cells, *iii*- with both two-photon and confocal microscopes. To date, the biological applications of the red-edge effect have primarily concerned the membrane and protein science^29^,^39^, owing to their high level of structural organization and confinement. The discovery of higher-order DNA and RNA structures (chiefly quadruplexes) with well-defined three-dimensional binding pockets, bridges the protein and nucleic acid research fields, suggesting that small molecules that bind to quadruplexes might interact with protein-like hydrophobic binding sites in which REE is possible. We demonstrated here REE is indeed occurring in the nucleic acid environment, using a TASQ ligand as a REE probe to assess the existence of both DNA and RNA quadruplexes in human cells by fluorescence cell imaging. This study, performed in both *in vitro* conditions and cellular context, highlights the advantages of the red-edge excitation over the conventional fluorescence approach, primarily the flexibility in fluorescent wavelength selection for *ad hoc* experimental setups (selection of excitation/emission wavelengths, of microscope facilities, etc.). We believe that, given the aromatic nature of most cellular targets and fluorescence dyes routinely used for cellular imaging purposes, the REE might be a more general phenomenon that initially anticipated, especially for confocal analyses where mounting agents (such as Fluoromount-G used here) might favor REE responses by increasing the local confinement and viscosity of prepared cells. We nevertheless recognize that searching for a fluorescence response where fluorophores do not absorb light is counter-intuitive enough not to be systematically investigated, but this study stresses the importance of looking for REE responses when possible, since it might provide alternative and complementary information without adding extra steps to ongoing cellular imaging experiments. Of course, some limitations exist, notably in terms of sensitivity of detection, since REE probes provide low-intensity responses or, as encountered above, for co-localization experiments with other fluorophores, since REE probes provide signal trough every emission filters. However, recent technological advances (high-sensitivity cameras and detectors) and practical consideration (difference in quantum gain) allow for addressing most of these issues. At present, we do not know the extent to which the red-edge effect –and the corresponding REE probes– will find applications in chemical biology quests; we contended that the new quadruplex-specific REE probe presented here open up avenues for the design of more versatile and efficient quadruplex-reporting dyes for fluorescence cell imaging.

## Experimental section

### Materials

All compounds studied here (N-TASQ, N-NH2 and ^PNA^DOTASQ) have been prepared according to published protocols (Ref. 18,23). BRACO-19 was purchased from Sigma-Aldrich and used without further purification.

### Oligonucleotides

Lyophilized DNA/RNA oligonucleotides were purchased from Eurogentec (Seraing, Belgium). Oligonucleotides were firstly diluted at 500 μM in deionized water. Quadruplex structures (22AG, c-Myc and TERRA) were subsequently prepared in a buffer comprising 10 mM lithium cacodylate buffer (pH 7.2) plus 10 mM KCl/90 mM LiCl, by mixing 40 μL of the constitutive strand (500 μM in H_2_O) with 8 μL of a lithium cacodylate buffer solution (100 mM, pH 7.2), plus 8 μL of a KCl/LiCl solution (100 mM/900 mM) and 24 μL of water. Duplex structure (ds17) was prepared by mixing 40 μL of each constitutive strand (500 μM) with 16 μL of a lithium cacodylate buffer solution (100 mM, pH 7.2), plus 16 μL of a KCl/LiCl solution (100 mM/900 mM) and 48 μL of water. The precise oligonucleotide concentrations were determined *via* UV spectral analyses of solution at 1 μM theoretical concentration at 260 nm after 5 min at 90 °C with the following molar extinction coefficient values: 228500, 232000, 223400 and 328300 M^−1^.cm^−1^ for 22AG, c-Myc, TERRA and ds17, respectively.

### REE experiments

Emission and excitation spectra were recorded on a JASCO FP8500 spectrofluorimeter in a 10 mm path-length quartz semi-micro cuvette (Starna). Experiments were carried out in 1 mL (final volume) of 10 mM lithium cacodylate buffer (pH 7.2) + 90 mM LiCl/10 mM KCl, without or with N-TASQ (10 μM, 2 mM stock solution in H_2_O, 5 μL) alone or in presence of DNA/RNA oligonucleotides (22AG, c-Myc, TERRA or ds17, up to 5 μM, 1 μM pitch). Emission spectra were recorded at various λ_ex_ (408, 488, 555 nm, and between 488–588 nm or 618–638 nm, 10 nm pitch); excitation spectra were recorded at various λ_em_ (634–734 nm), with the following settings: Ex bandwidth = 5 nm, Em bandwidth = 10 nm, 1 nm pitch, 1 s response, high sensitivity, scan speed = 500 nm.mn^−1^, at 20 °C.

### Cell culture

MCF7 cells were routinely cultured in 75 cm^2^ tissue culture flasks (Corning) at 37 °C in a humidified, 5% CO_2_ atmosphere in Dulbecco’s Modified Eagle Medium (DMEM, Gibco) supplemented with 5% fetal bovine serum (FBS, Gibco) and 100U Penicillin-Streptomycin (Pen-Strep, 1.0 u.mL^−1^ Pen/1.0 μg.mL^−1^ Strep, Gibco) mixture. Cells were subcultured twice weekly using standard protocols: removal of the medium, PBS washing step, incubation with Trypsin-EDTA (0.25%, Gibco) 5 min at 37 °C; cells were subsequently manually harvested, counted (Beckman Coulter) and reseeded in appropriate density.

### Cellular imaging

First cellular images were performed with MCF7 cells seeded on round glass coverslips and allowed to recover for 24 h. For RNA quadruplex visualization: MCF7 cells were incubated with N-TASQ (2.5 μM) for 24 h. Cells were washed with PBS then fixed in 3.7% paraformaldehyde (w/v) for 15 min and again washed with PBS then permeabilized with 0.1% Triton X-100 in CSK buffer. Following 3 thorough washing with PBS, cells were mounted with Fluoromount-G prior to cellular imaging. For DNA quadruplex visualization: MCF7 cells were incubated with BRACO-19 (0–8.4 μM) for 24–48 h. Cells were washed 1x with PBS then fixed and permeabilized with 100% MeOH for 10 min at 25 °C. Coverslips were washed with PBS, and incubated with 2.5 μM or 100 μM N-TASQ for 3 h at 25 °C. Following 3 washing with PBS and cells were mounted. N-TASQ/BG4 co-localization experiments were realized as follows: cells were seeded onto round glass coverslips in 24-well plates (Corning), allowed to recover for 24 h. Cells were washed 1x with PBS then fixed in 3.7% paraformaldehyde (w/v) for 15 min and again washed 2x in PBS then permeabilized with 0.1% Triton X-100 in 1xCSK buffer. Alternatively, cells were fixed and permeabilized with 100% MeOH for 10 min at room temperature. Coverslips were washed 3x with PBS then incubated for 1 h with 4% BSA block, and 10 μg/ml BG4 was added and allowed to label for at least 3 h at room temperature. Following BG4 binding, coverslips were washed with PBS (3 times), incubated with secondary antibodies (2Ab) against Flag tag (Sigma) for 3 h at room temperature. Coverslips were then washed with PBS (3 times), incubated with anti-mouse antibodies conjugated with AF594 (AF594-3Ab, Molecular Probes) for 3 h at room temperature. Coverslips were subsequently washed with PBS (3 times), and incubated with 2.5 μM or 100 μM N-TASQ for 3 h at room temperature. Control experiments are performed with 2Ab and AF594-2Ab incubations only (Figure S10). Following 3 thorough washings with PBS, cells were mounted with Fluoromount-G. Confocal images were recorded using a Zeiss LSM 700 confocal laser scanning microscope and ZEN software (Carl Zeiss Microimaging, Gottingen, Germany), using DAPI, AF488 and AF594 filters (lasers at 408, 488 and 555 nm, and the tracks for each channel: 495 and below, 495–590 and 585 nm and above). Control experiments conducted with a Nikon C1si/PicoQuant confocal laser scanning microscope can be found as Supplementary Material (Figure S11).

## Additional Information

**How to cite this article**: Laguerre, A. *et al.* Direct visualization of both DNA and RNA quadruplexes in human cells *via* an uncommon spectroscopic method. *Sci. Rep.* **6**, 32141; doi: 10.1038/srep32141 (2016).

## Supplementary Material

Supplementary Information
