# Supplementary material for: Direct visualization of both DNA and RNA quadruplexes in human cells via an uncommon spectroscopic method
Source: Sci Rep. 2016 Aug 18;6:32141. doi: 10.1038/srep32141 (PMC4989495; doi:10.1038/srep32141)
Supplement: Supplementary Information [file srep32141-s1.pdf]

Direct visualization of both DNA and RNA quadruplexes in human cells *via* an uncommon spectroscopic method

Aurélien Laguerre,[1] Judy M. Y. Wong,[2,*] and David Monchaud[1,*]

[1]Institut de Chimie Moléculaire, ICMUB CNRS UMR6302, UBFC Dijon, France.

[2]Faculty of Pharmaceutical Sciences, The University of British Columbia, Vancouver, Canada.

-- Supporting Information --

I. Theory of the Red-Edge Effect .... Page S1
II. Confirmation of REE with TERRA .... Page S3
III. REE with other oligonuculeotides .... Page S3
IV. Cellular investigations .... Page S5

I. Theory of the Red-Edge Effect

Fluorescence, in its broadest definition, is governed by a substantial number of rules. Among them, the Vavilov's rule stipulates that "*the quantum yield of luminescence is independent of the wavelength of exciting radiation*" (IUPAC definition). This rule thus explains that an irradiation at any wavelength within the spectral width defined by the absorption spectrum of a fluorophore provides an emission centered on a single wavelength (only the emission intensity varies according to the molar absorptivity corresponding to the selected irradiation wavelength). Absorption spectra reflect the transition from the ground-state energy level S_0 to the excited-state level S_1. However, as seen in the Jablonski diagram depicted in Figure S1A, each energy level comprises additional energy sublevels that correspond to vibrations of the molecule in its ground/excited states, consequently termed vibrational levels and defined by the Franck-Codon principle. The presence of these additional sublevels makes the $S_0 \rightarrow S_1$ transition possible through various excitation energies (E_a, E_b and E_c), that is, through various excitation wavelength (λ_a, λ_b and λ_c) according to the Planck's equation ($E = h\nu = hc/\lambda$, with h stands the Planck's constant and ν, c and λ for the frequency, speed and wavelength of the electromagnetic radiation, respectively). Therefore, absorption spectra are the sum of a set of transition energies (E_a, E_b.. E_n), but individual contribution cannot be distinguished, making absorption spectra usually poorly-defined: this is called the *homogeneous broadening*.

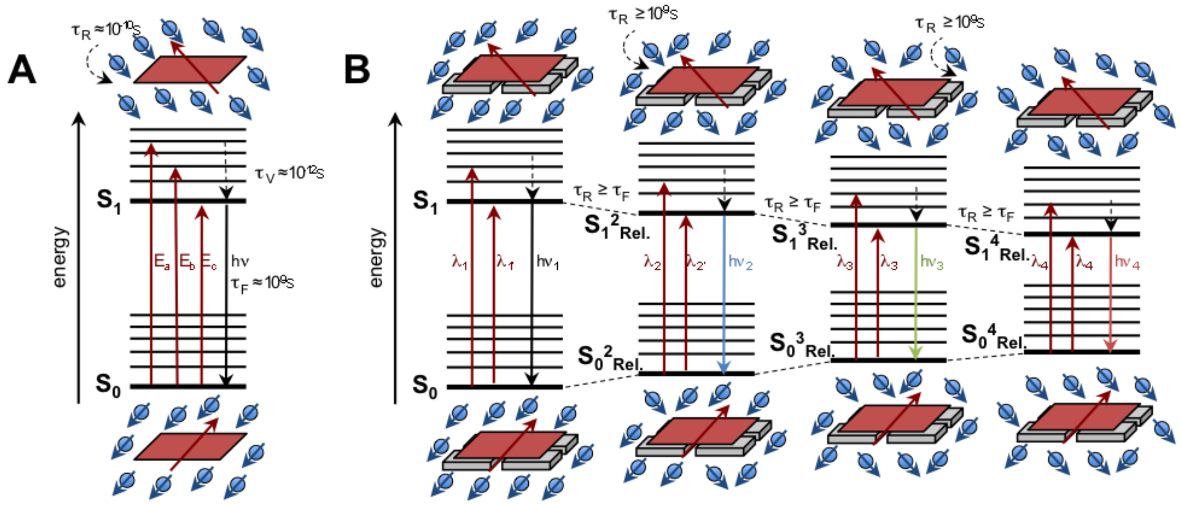

Figure S1. A, Jablonski diagram depicting the ground (S_0) and excited states (S_1) of a fluorophore (red rectangle) in dilute conditions, in which solvent (blue circles) relaxation occurs more rapidly ($\tau_R \approx 10^{-10}$s) than light emission ($\tau_F \approx 10^{-9}$s). **B**, Jablonski diagram corresponding to the same fluorophore in a constrained environment (here, in interaction with a G-quartet) in which solvent relaxation is slowed down ($\tau_R \geq \tau_F$): this condition creates a continuous model of solvent relaxation, characterized by intermediate ground ($S_0{}^{n}{}_{Rel.}$) and excited states ($S_1{}^{n}{}_{Rel.}$), leading to intermediate excitation and emission wavelengths that account for REE.

The Vavilov's rule is pursuant to the Kasha's rule, which stipulates that photon emission occurs only "*from the lowest excited state of a given multiplicity*" (IUPAC definition), that is, that the $S_1 \rightarrow S_0$ transition occurs only from the lowest energy sublevel of S_1, accessed through vibrational relaxation (dashed arrows, Figure S1A). However, some exceptions to the classical applications of the Kasha-Vavilov rules exist. In dilute solution, the $S_1 \rightarrow S_0$ transition (emission) usually occurs in the 10^{-9} to 10^{-6}-s time scale (τ_F), that is, slower than the solvent relaxation ($\tau_R \approx 10^{-10}$ s) that leads to an alignment of solvent dipoles opposite to the fluorophore's dipole to decrease the interaction energy (Figure S1A). In highly ordered environments, solvent mobility is restricted and solvent relaxation becomes slower, that is, $\tau_R \geq \tau_F$: this leads to various intermediate states between the initial (S_1) and the final, relaxed excited state ($S_{1Rel.}$), with partially aligned solvent dipoles (Figure S1B). This phenomenon is of critical importance in polar environments in which dipole-dipole interactions have significant contributions; gradual solvent relaxation lowers the energy level of each intermediate, thus decreasing the $S_0{}^{n}{}_{Rel.} \rightarrow S_1{}^{n}{}_{Rel.}$ transition cost that could be achieved with photons of lower energy, *i.e.*, with red-shifted absorbance wavelengths (λ_n). Therefore, absorption spectra of fluorophore in constrained environments are the sum of a set of new and lower transition energies (E_1, E_2.. E_x), but individual contribution cannot be distinguished,

making the absorption poorly-defined on the red edge of its spectrum: this is called the *inhomogeneous broadening*. Inhomogeneous broadening is thus responsible for the REE; this effect does not violate the Kasha-Vavilov rules *per se*, it solely originates in their application in very specific conditions.

II. Confirmation of REE with TERRA.

The REE is characterized by both the dependence of the wavelength of the emission maximum on the excitation wavelength, $\lambda_{em}^{max} = f(\lambda_{ex})$, and the reciprocal dependence of the wavelength of the excitation maximum on the emission wavelength, $\lambda_{ex}^{max} = f(\lambda_{em})$. Initial demonstration was performed with N-TASQ (10μM) and a DNA quadruplex (22AG, d[$^{5'}$AG$_3$(T$_2$AG$_3$)$_3$$^{3'}$], 5μM) (cf. the main article). To confirm the generality of these observations, similar titrations were performed with N-TASQ (10μM) and a RNA quadruplex (TERRA, r[$^{5'}$G$_3$(U$_2$AG$_3$)$_3$$^{3'}$], 5μM). As seen in Figure S2, the correlation between wavelengths of excitation (λ_{ex}) and emission (λ_{em}^{max}) is linear, with a strong fit (R^2 = 0.99713) and a slope of 0.81545 that highlights that Stokes shift gradually decreases (from 104 to 76nm) when the λ_{ex} increase towards the red region.

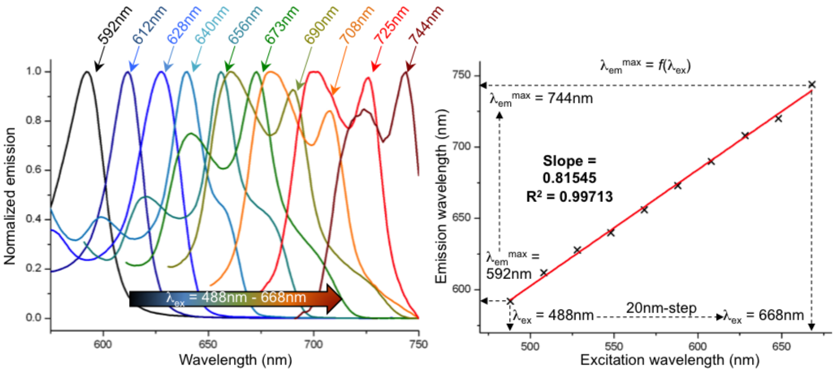

Figure S2. Dependence of the emission maximum (λ_{em}^{max}) on the excitation wavelength (λ_{ex}): fluorescence emission spectra recorded for an experiment carried out with N-TASQ (10μM) and TERRA (5μM) with λ_{ex} between 488 and 668nm (every 20nm) in 10mM lithium cacodylate buffer (pH 7.2) + 10mM KCl/90mM LiCl.

III. REE with other oligonucleotides.

a/ Titrations with 22AG, c-Myc, TERRA and ds17: the emission pattern of N-TASQ in interaction with 22AG fits within the emission filters routinely used with confocal microscopes (DAPI: 495nm and below; FITC: 495-590nm; Alexa: 585nm and above, cf. the main article). To confirm the generality of these observations, titrations were performed

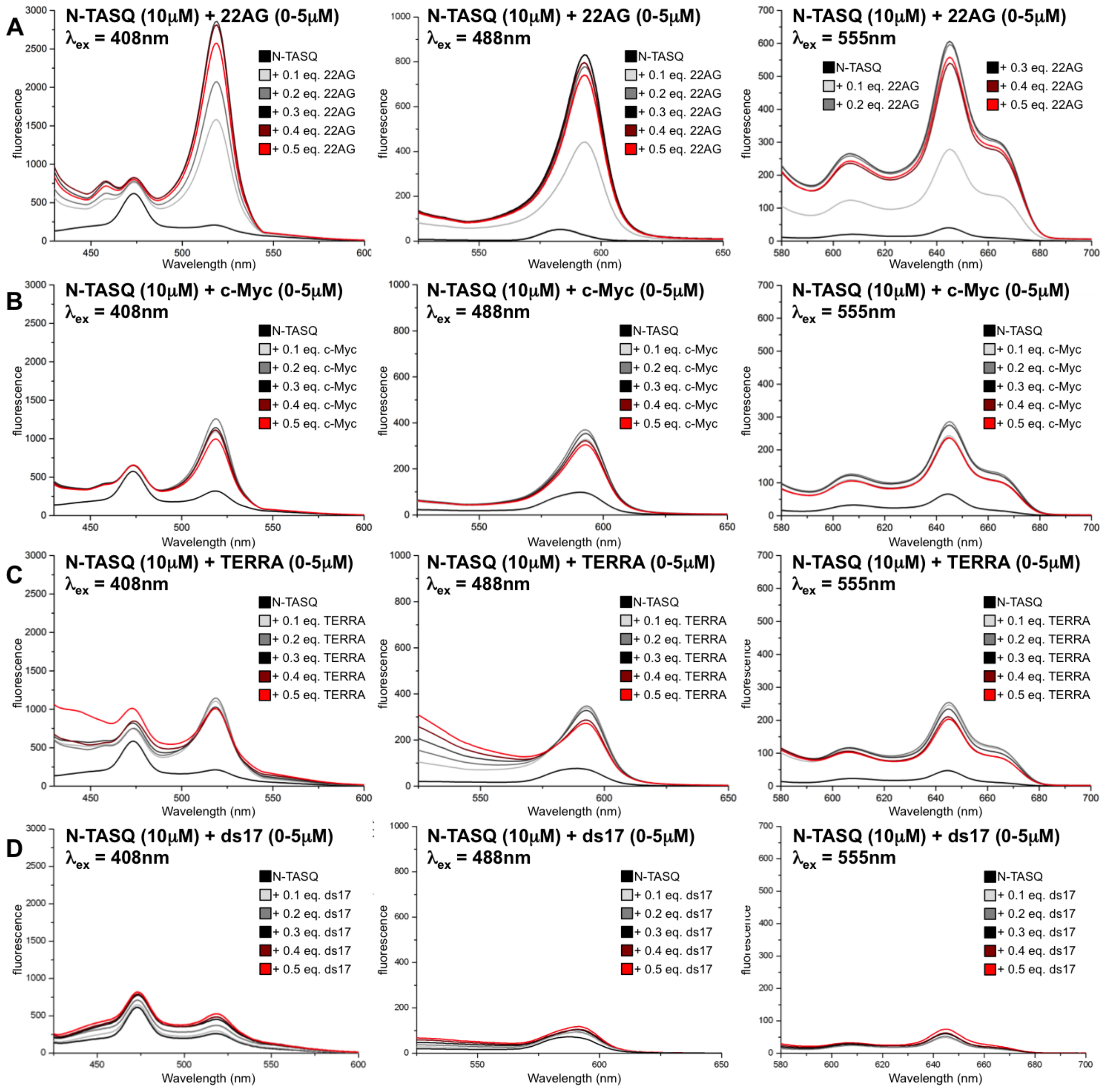

Figure S3. Fluorescence titrations performed without (black lines) or with N-TASQ (10µM) and increasing amounts of oligonucleotides (from 0 to 5µM, from grey to red lines), either quadruplex-DNA (22AG, **A** or c-Myc, **B**), quadruplex-RNA (TERRA, **C**) or duplex-DNA (ds17, **D**) at λ_{ex} = 408, 488 and 555nm, in 10mM lithium cacodylate buffer (pH 7.2) + 10mM KCl/90mM LiCl.

with N-TASQ (10µM) and increasing amounts (0 to 5µM) of quadruplex-DNA (22AG, see above, and c-Myc, d[$^{5'}GAG_3TG_4AG_3TG_4A_2G^{3'}$]), quadruplex-RNA (TERRA, see above) and duplex-DNA (ds17, d[$^{5'}C_2AGT_2C(GTA)_2AC_3^{3'}$]/d[$^{5'}G_3T(TAC)_2GA_2CTG_2^{3'}$]) under excitation at wavelengths that correspond to lasers of standard confocal microscopes, that is, λ_{ex} = 408, 488 and 555nm, in 10mM lithium cacodylate buffer (pH 7.2) + 10mM KCl/90mM LiCl. As seen in Figure S3, the N-TASQ emission maxima at 474, 519, 592, 607 and 647nm increase only upon interaction with DNA/RNA quadruplexes (and not with duplex-DNA); they

consequently confirm the suitability of N-TASQ for the visualization of both DNA and RNA quadruplexes by confocal microscopy.

b/ Reverse titrations with c-Myc: to confirm also the generality of the fluorescence response, reverse titrations were subsequently performed with c-Myc (10µM) and increasing amounts (0 to 10µM) of N-TASQ under excitation at wavelengths that correspond to lasers of standard confocal microscopes, that is, λ_{ex} = 408, 488 and 555nm. As seen in Figure S4, the N-TASQ emission maxima at 474, 519, 592, 607 and 647nm increase in a dose-response manner, thus confirming again the suitability of N-TASQ for the quadruplex detection by confocal microscopy.

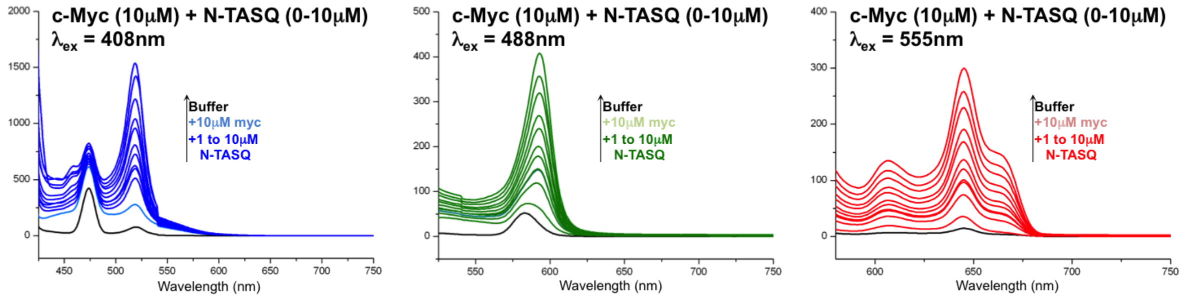

Figure S4. Reverse fluorescence titrations performed without (black lines) or with c-Myc (10µM) and increasing amounts of N-TASQ (from 0 to 10µM, colored lines), at λ_{ex} = 408, 488 and 555nm in 10mM lithium cacodylate buffer (pH 7.2) + 10mM KCl/90mM LiCl.

IV. Cellular investigations.

a/ Toxicity of N-TASQ: the visualization of RNA quadruplexes could be readily performed *via* live cell incubation of N-TASQ; however, sublethal concentrations must be used and as described in the main article, we used 2.5µM since N-TASQ does not display toxicity against MCF7 in a concentration range between 1.1 and 32.7µM for 120h treatments.

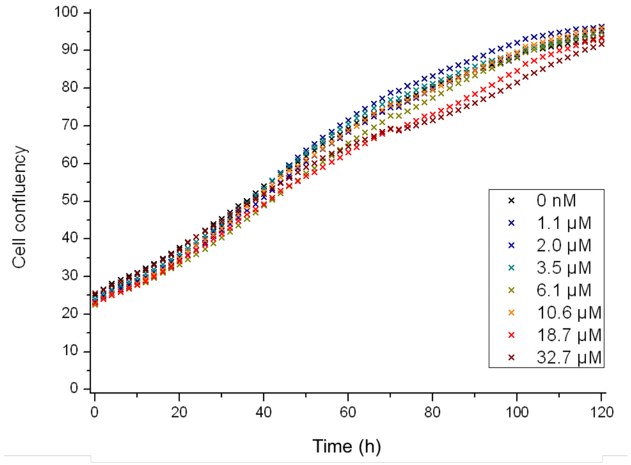

Figure S5. N-TASQ toxicity against MCF7 cells.

b/ Control experiments carried out with 2.5 *versus* 100µM N-TASQ as post-staining agent: to further support the versatility of N-TASQ as REE probes (and also in line with the live-cell experiments performed with cells incubated with 2.5µM N-TASQ for 24h) we investigated if post-fixation staining can be carried out with lower N-TASQ concentration (MeOH fixation).

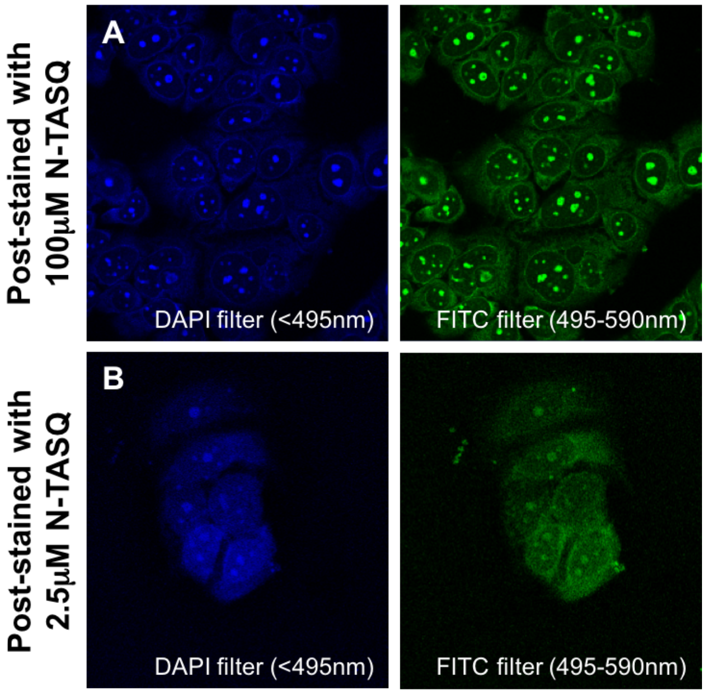

As seen in Figure S6A, using 2.5µM N-TASQ as post-staining agent provides good images, with a labelling pattern in agreement with the one obtained using 100µM N-TASQ (Figure S6B) albeit with a weaker fluorescence intensity (in line with the lower concentration of the staining agent).

Figure S6. Confocal images of MCF7 cells labeled after MeOH fixation with either 100 µM (A) or 2.5µM N-TASQ (B).

c/ Control experiments carried out with 2.5µM N-TASQ as post-staining agent and increasing amounts of BRACO-19 (0 to 5µg/mL): having established (point b/ above) that 2.5µM N-TASQ is useful as a post-staining dose for MeOH-fixed MCF7 cells, we performed BRACO-19 dose-response experiments, treating MCF7 cells with 0 to 5µg/mL for 48h before cell fixation (MeOH) and post-fixation labelling with 2.5µM N-TASQ. As seen in Figure S7, using a lower concentration of N-TASQ (2.5µM) as post-stain allows for the observation of an increase in G-quadruplex formation with BRACO treatment at low concentrations (0.625µg/mL, Figure S7A *versus* S7B) but a loss of N-TASQ staining at higher dose (1.25-5µg/mL, Figures S7C,D), likely due to the competition that takes place between the two quadruplex ligands.

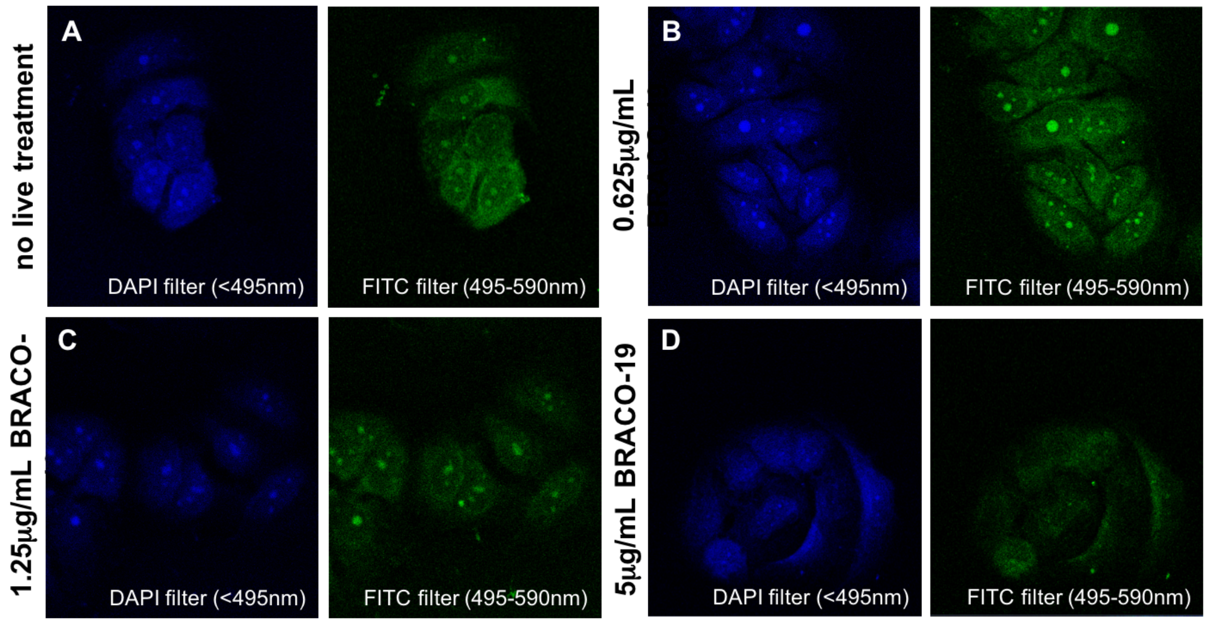

Figure S7. Confocal images of MCF7 cells untreated (A) or live-incubated with 0.625 (B), 1.25 (C) or 5µg/mL BRACO-19 (48h) and labeled after MeOH fixation with either 2.5µM N-TASQ.

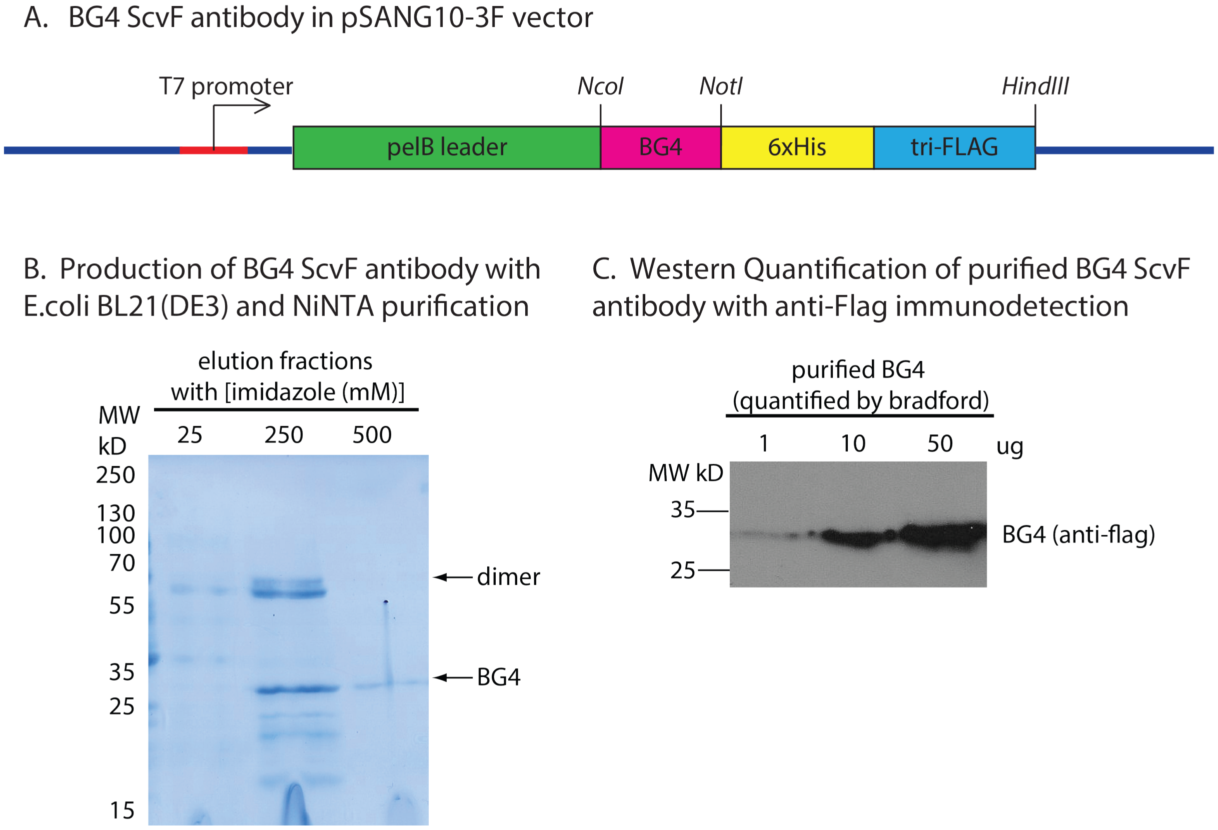

Figure S8. Schematic representation of the production and purification protocols of BG4 antibody.

d/Preparation of BG4: We expressed the BG4 ScvF antibody (schematic of expression vector shown in Figure S8) in E. coli BL21(DE3) strain under ampicillin selection. Following cell lysis by sonication, BG4 purification was performed through a single round of NiNTA resin binding and elution. Purified fractions were separated by SDS-PAGE and visualized with coomassie blue staining (B). Single peptide, corresponding to the expected molecular weight of BG4 was recovered in eluate fraction 2 (at 250mM imidazole). We also detected a peptide signal corresponding to BG4 dimer. This dimer signal resolved (disappeared) after dialysis against storage buffer. Purified BG4 ScvF antibody was quantified with standard Bradford protein assay and visualized by anti-flag western detection (C).

e/ Labelling MCF7 with N-TASQ, BG4 or both: REE stains provide fluorescence outputs in every emission filters during confocal analysis; in case of co-staining experiments with other fluorophores, this capacity can represent a limitation to the broad applicability of REE probes. This issue can be conveniently addressed on the basis of the difference between the quantum gains of REE probes *versus* conventional fluorophores. Here, we have stained fixed MCF7 cells with either N-TASQ (100μM), or BG4 (detected through incubation with a secondary antibodies conjugated to an Alexa594 probe), or both concomitantly. As seen in Figure S9A,B, images collected upon irradiation at 555nm and collected through the Alexa filter (585nm and above) show that the pattern obtained in the presence of both N-TASQ and BG4 (right panels) correspond to that of BG4 alone (center panels) only, owing to the weak emissive nature of REE probes. This highlight that co-staining experiments can be conveniently performed with both REE and conventional stains. It should be noted that BG4 results are somewhat surprising in MeOH-fixed cells, since nucleoli seems to be strongly labeled while such staining has not been reported in the two initial articles (G. Biffi *et al.*, *Nature Chem.* **2013**, *5*, 182 & *Nature Chem.* **2014**, *6*, 75). This was further investigated *via* two-photon investigations that provides higher-resolution images (experiments were performed on a Nikon A1-MP scanning microscope (Nikon, Japan) with a ×60 Apo LWD objective (NA: 1.27, Water Immersion, Nikon, Japan)). As seen in Figure S9C,D, collected images confirm that BG4 labels quadruplexes mostly in cytoplasmic sites (RNA quadruplexes) after PFA-triton fixation and in nuclear sites (DNA quadruplexes) after MeOH fixation, and that bright foci seen in Figure S9B (center and right panel) actually correspond to isolated nuclear foci and not to nucleoli labelling.

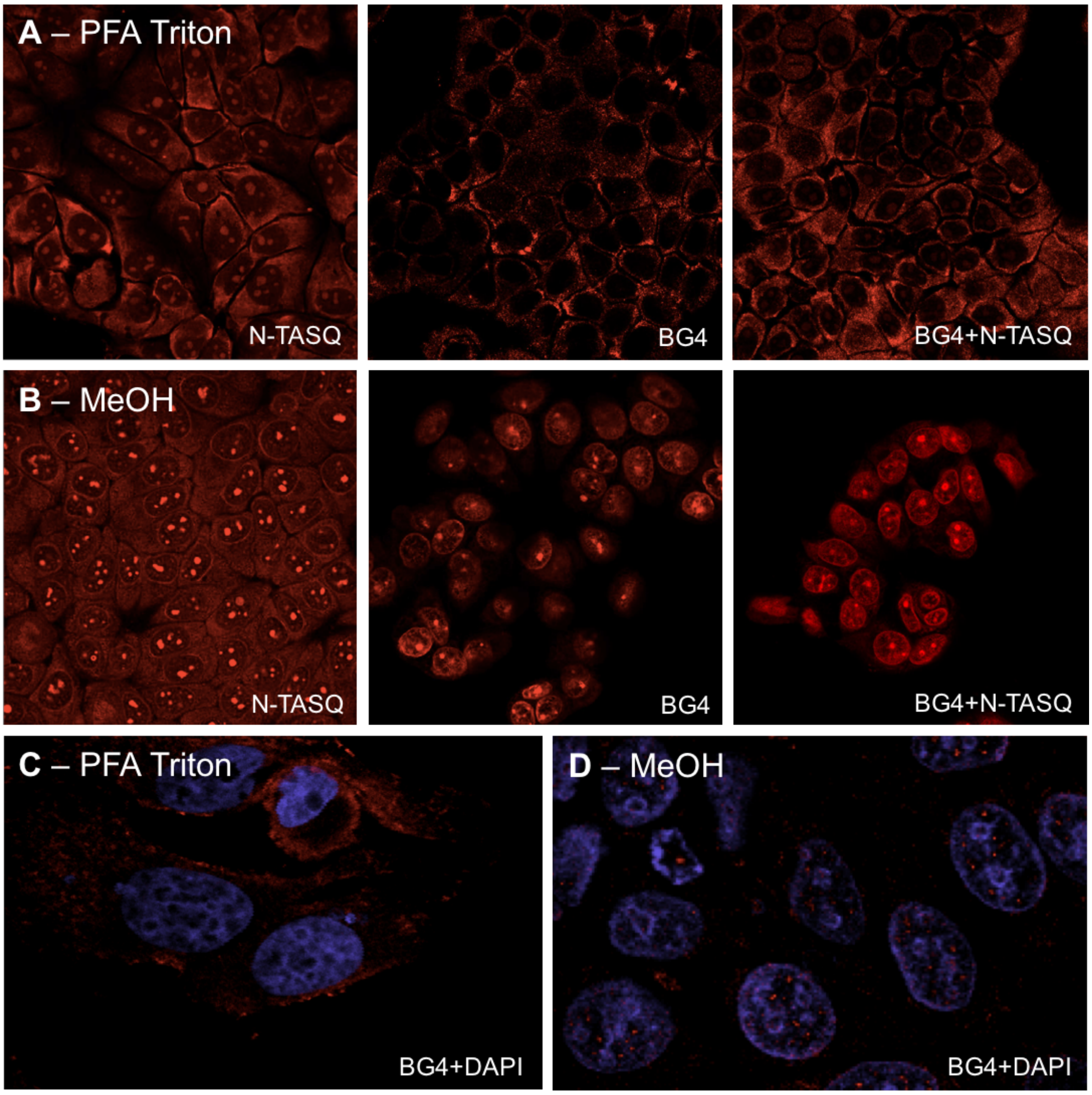

Figure S9. A,B Fixed MCF7 cells (with PFA-triton (**A**) or MeOH (**B**) as fixative) post-labeled with N-TASQ (100μM, left panel), BG4 (10μg/mL, center panel) or both (right panel). Confocal analysis carried out with lasers at 555nm and visualized through Alexa channel. **C,D** Fixed MCF7 cells (with PFA-triton (**C**) or MeOH (**D**) as fixative) post-labeled with BG4 (10μg/mL, center panel) and counter-stained with DAPI. Two-photon images were collected on a Nikon A1-MP microscopes, with laser adjusted at 720nm and visualized through DAPI (FF01-492/SP) and Alexa (FF01-629/56) Semrock filters; only merged images are shown.

f/ Control experiments: having established (point c/ above) that the combination of 2.5μM N-TASQ/0.625μg/mL BRACO-19 provides suited conditions to observe DNA quadruplexes in an optimal manner in our experimental setup, we performed immunodetection control experiments labelling BRACO-19-treated cells by 1- N-TASQ alone (2.5μM), 2- N-TASQ and BG4 + AlexaFluor594-tagged secondary antibodies (referred to as "2Ab" and "AF-594-3Ab"), and 3- N-TASQ and AF-594-3Ab only. Conclusions drawn from the images seen in Figure S10 are the following: 1- this series of images confirms that the DAPI and FITC channels are completely reflective of N-TASQ staining, while the Alexa channel is not (Figure S10A); 2- the

labelling pattern seen in Figure S10B is in agreement with the one seen in Figure 5B (main manuscript) albeit with a lower contrast due to the lower N-TASQ concentration used herein; 3- this series of images also shows that BG4 incubation precludes efficient N-TASQ labelling when it is used at low concentrations possibly due to a competition between the antibody and the ligand (Figure S10A versus S10B); and 4- that the low-intensity red cytoplasmic background is an artefact of the 2AB+AF594-3Ab antibody *per se* (Figure S10C). Collectively, these experiments demonstrate that N-TASQ and BG4 can be used concomitantly in a reliable manner and beyond this, that they have similar cellular targets therefore lending credence to the capability of N-TASQ to detect quadruplexes cells.

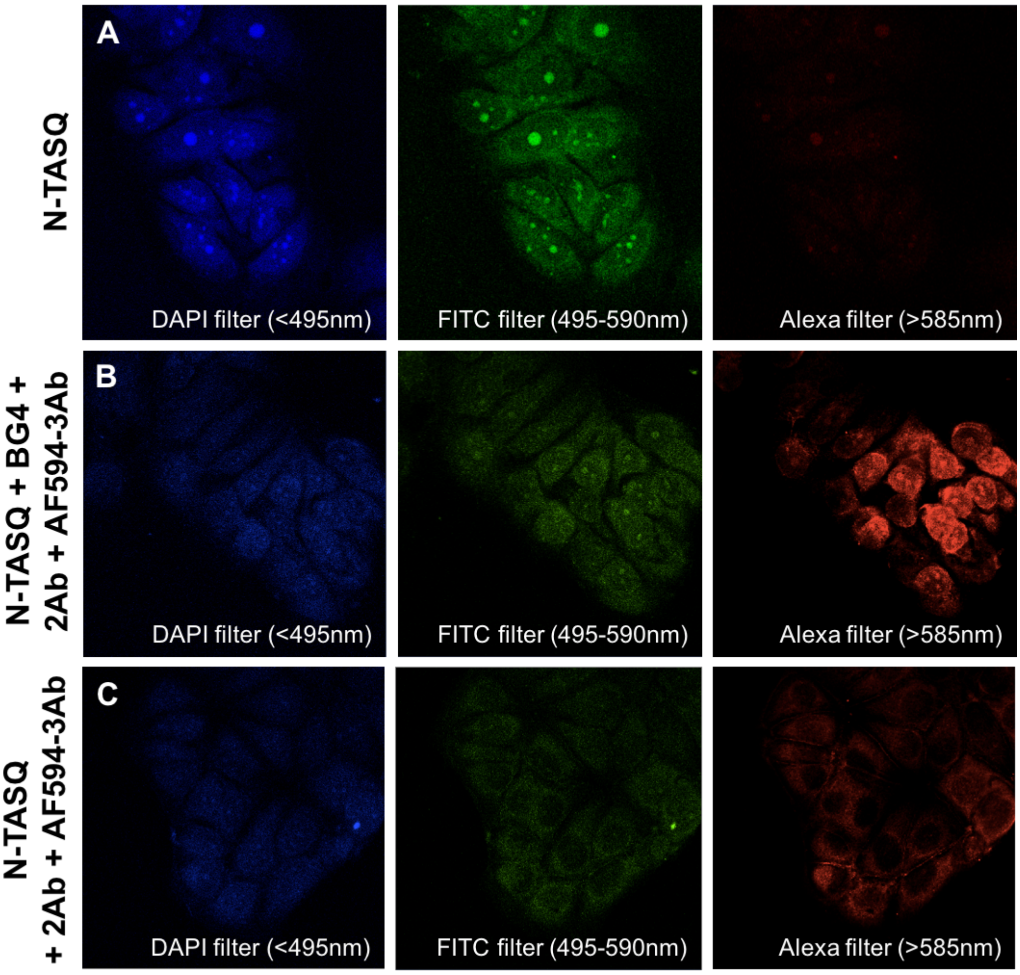

Figure S10. Confocal images of MCF7 cells live-incubated with 0.625μg/mL BRACO-19 (48h) and labeled after MeOH fixation with either 2.5μM N-TASQ (A), N-TASQ, BG4 and its secondary, labeled antibodies (2AB and AF594-3Ab, B), or N-TASQ and only the labeled antibody 2AB+AF594-3Ab (C).

g/ N-TASQ as REE probe control experiments: to investigate whether N-TASQ might be a REE probe whatever the nature of both the confocal microscope and the cell line, a series of control images were collected with a Nikon C1si/PicoQuant confocal (*versus* a Zeiss LSM 700 used in the main manuscript) using N-TASQ as post-labelling agent on fixed HT29 colorectal cancer cells (*versus* MCF7 breast cancer cells used in the main manuscript). Collected images confirm the suitability of N-TASQ as REE probes with alternative imaging facilities and cell lines.

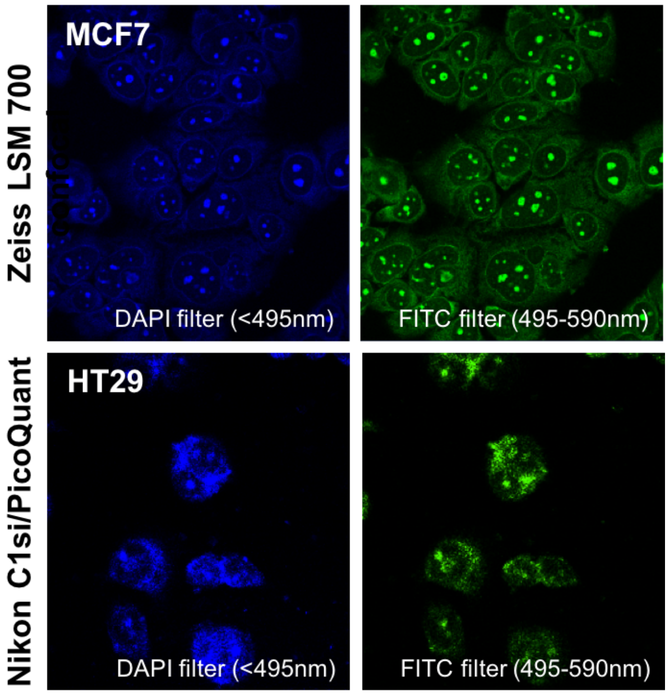

Figure S11. N-TASQ acts as a REE probe with two cell lines (MCF7 and HT29) and two microscopes (Zeiss LSM 700 and Nikon C1si/PicoQuant).
